# Burden of influenza, respiratory syncytial virus, and other respiratory viruses and the completeness of respiratory viral identification among respiratory inpatients, Canada, 2003‐2014

**DOI:** 10.1111/irv.12497

**Published:** 2017-12-15

**Authors:** Dena L. Schanzer, Myriam Saboui, Liza Lee, Andrea Nwosu, Christina Bancej

**Affiliations:** ^1^ Infectious Disease Prevention and Control Branch Public Health Agency of Canada Ottawa ON Canada

**Keywords:** completeness of case ascertainment, disease burden, influenza‐attributed hospitalization rates, respiratory syncytial virus, respiratory viral identification, severe acute respiratory infections

## Abstract

**Background:**

A regression‐based study design has commonly been used to estimate the influenza burden; however, these estimates are not timely and many countries lack sufficient virological data. Alternative approaches that would permit a timelier assessment of the burden, including a sentinel surveillance approach recommended by the World Health Organization (WHO), have been proposed. We aimed to estimate the hospitalization burden attributable to influenza, respiratory syncytial virus (RSV), and other respiratory viruses (ORV) and to assess both the completeness of viral identification among respiratory inpatients in Canada and the implications of adopting other approaches.

**Methods:**

Respiratory inpatient records were extracted from the Canadian Discharge Abstract Database from 2003 to 2014. A regression model was used to estimate excess respiratory hospitalizations attributable to influenza, RSV, and ORV by age group and diagnostic category and compare these estimates with the number with a respiratory viral identification.

**Results:**

An estimated 33 (95% CI: 29, 38), 27 (95% CI: 22, 33), and 27 (95% CI: 18, 36) hospitalizations per 100 000 population per year were attributed to influenza, RSV, and ORV, respectively. An influenza virus was identified in an estimated 78% (95% CI: 75, 81) and 17% (95% CI: 15, 21) of respiratory hospitalizations attributed to influenza for children and adults, respectively, and 75% of influenza‐attributed hospitalizations had an ARI diagnosis.

**Conclusions:**

Hospitalization rates with respiratory viral identification still underestimate the burden. Approaches based on acute respiratory case definitions will likely underestimate the burden as well, although each proposed method should be compared with regression‐based estimates of influenza‐attributed burden as a way of assessing their validity.

## 1 INTRODUCTION

Despite significant efforts to assess the burden and severity of the 2009 pandemic strain (A(H1N1)pdm09) in real time, several gaps in global influenza surveillance capacity became apparent, notably the completeness of case ascertainment, which the World Health Organization (WHO) believed to be quite low globally.^1^ In response, the WHO issued a briefing note in 2009 advising that statistical models similar to the methodology introduced by Thompson et al in 2003^2^ are required to more completely estimate the burden associated with influenza,^1^ and later funded an international study to use a statistical model to estimate the global burden associated with A(H1N1)pdm09.^3^ Subsequently, to facilitate real‐time assessment of the burden, enhance influenza surveillance globally, and promote standardization, the WHO developed influenza surveillance guidelines in consultation with member countries.^4^

Since 2003, a regression approach has been widely used in many countries,^3^, ^5^ including Canada to estimate the excess burden associated with influenza and other respiratory viruses.^6^, ^7^, ^8^, ^9^ Because regression estimates are not available in real time, researchers have proposed other methods^10^, ^11^ to use surveillance data to predict the full burden in near real time, only some of which have been evaluated at year‐end against a regression approach.^6^, ^12^, ^13^, ^14^

Despite the focus on developing methods for more timely estimates of the burden associated with influenza, other respiratory viruses cause considerable disease burden in Canada.^15^, ^16^ In this study, our objectives were (i) to update our estimates of the average annual respiratory hospitalization rate attributable to influenza, RSV, and other respiratory viruses in Canada by age group and disease severity; (ii) to quantify the degree to which the rates of hospitalizations coded as due to those virus rates underestimate this burden; and (iii) to discuss issues related to adopting emerging approaches based on near real‐time data that would permit more timely assessment of the burden of influenza and other respiratory viruses. The proportion of hospitalizations attributed to influenza and other respiratory viruses for which the respiratory virus was identified provides an approximate measure of completeness of viral identification, estimates that help hospital administrators interpret their data, as the corresponding multiplier (reciprocal of completeness) has been used in this way with near real‐time surveillance data.^10^ As estimation of the hospitalization burden attributable to influenza for the pediatric population has been a challenge, we explored this issue by comparing the number of admissions with the influenza virus identified to the estimated excess attributed to influenza for various diagnostic groups. We also assessed the potential impact of restricting testing to various clinical case definitions on the burden estimates.

## 2 METHODS

### 2.1 Data source

Hospital discharge records for all patients admitted to an acute care hospital for a respiratory condition between September 2003 and August 2014 were extracted from the Canadian Institute of Health Information (CIHI) patient‐specific Discharge Abstract Database (DAD).^17^ The province of Quebec does not participate in the DAD;^17^ hence, the DAD includes approximately 75% of all acute care hospital separations in Canada. The study period included 9 influenza seasons defined as running from September to the end of August of the following year, as we excluded the 2008/09 and 2009/10 influenza seasons due to the circulation of the A(H1N1)pdm09 strain. Throughout this period, the *International Classification of Disease, Tenth Modification* (ICD‐10),^18^ was used for coding diagnoses. Records were aggregated to weekly intervals and stratified by age group (<2 years; 2‐4 years; 5‐16 years; 17‐44 years; 45‐64 years; 65+ years), discharge status (discharged alive or dead), and severity (admittance to a special care unit [SCU] or mechanical ventilation) with nested diagnostic groups. (SCU is the term used by CIHI and may be known elsewhere as a high dependency unit, intensive care unit, or critical care unit.)

Denominators for rate calculations by age group and participating provinces were obtained from census and intercensual projections published by Statistics Canada.^19^ National estimates of the number of hospitalizations, where provided, were prorated for the Canadian population.

### 2.2 Measures of viral activity

Hospitalizations with an identified respiratory virus were classified into 3 viral groups: influenza (J10), RSV (J12.1, J20.5, J21.0, B97.4), and ORV (J12.0, J12.2‐J12.8, J20.0‐J20.4, J20.6‐J20.7, J21.1, B34.0‐B34.4, B97.0‐B97.3, B97.5‐B97.7) (see Appendix S1 for a description of these codes) and aggregated into 3 weekly time series to be used as the independent variables in each regression model. Viral identification in hospitalized patients could be from either a laboratory report or a point‐of‐care test.

### 2.3 Creation of the dependent variables for the regression model

A weekly time series was created for each subgroup for which we sought to estimate the excess burden associated with influenza, RSV, and ORV activity. Records were aggregated accordingly by age group and diagnostic group, discharge status, and severity as required for estimation of the excess burden. Diagnostic categories included all respiratory conditions (J00‐J99) as a primary diagnosis and the following subcategories: primary pneumonia and influenza (PNI) (J10‐J18), primary influenza‐like illness or unspecified acute respiratory infection (J06.9, J11), primary respiratory with any mention of an acute respiratory condition (ARI) (J00‐J22, J44.0) (as either a primary or secondary ARI diagnosis), and primary respiratory without any mention of an ARI dx as either a primary or secondary diagnosis. We interpreted the term “SARI” from the WHO surveillance guidelines to correspond to the ICD‐10 diagnostic codes for either ILI (J11) or ARI (J00‐J22, J44.0), although the WHO SARI case definition has not been formally evaluated against ICD‐10.^4^ All diagnosis codes were determined after discharge.

### 2.4 Statistical analysis

The weekly number of hospitalizations (by date of admission) for each subgroup was modeled as a function of viral activity, seasonality, and trend using a quasi‐Poisson regression model similar to previously published estimates of the influenza burden in Canada (see Appendix S2 for details)^6^, ^7^ and internationally.^3^ The number of hospitalizations attributed to each virus was calculated for each week by subtracting the estimated baseline from the model‐predicted number of hospitalizations. A separate baseline was calculated for each virus by setting the independent variable representing viral activity to zero and using the same model to predict the number of admissions under the hypothetical absence of the virus. To clarify both the viral attribution and the subgroup, the term “influenza‐attributed respiratory hospitalizations” is used to correspond to the estimated number of excess respiratory hospitalizations associated with periods of influenza activity and that are likely due to influenza. Results are reported as crude rates for ease of international comparison.

Completeness of viral identification for each virus type was calculated as the number of hospitalizations with the virus identified divided by the estimated number of hospitalizations attributed to the virus. Confidence intervals for completeness estimates correspond to the uncertainty in the denominator. When the lower 95% CI of the estimated excess was less than the number with viral identification, the upper 95% CI of the completeness estimate was replaced with 100%. Negative estimates of excess hospitalizations can arise due to chance alone if the true (though unknown) number of hospitalizations due to the virus is less than the usual variation in the total number of hospitalizations. In this case, the negative estimate is usually not statistically significant. A negative excess in hospitalizations associated with influenza activity could also arise due to the fixed number of hospital beds with limited surge capacity and the resulting need to prioritize admissions. In a previous study, we found non‐respiratory hospitalizations were lower than the estimated baseline during periods of influenza activity.^15^ Various methodological issues related to the regression approach have been discussed elsewhere.^6^, ^15^ Analyses were performed with sas (version 9.1; Cary, NC, USA).

## 3 RESULTS

**Figure 1:**
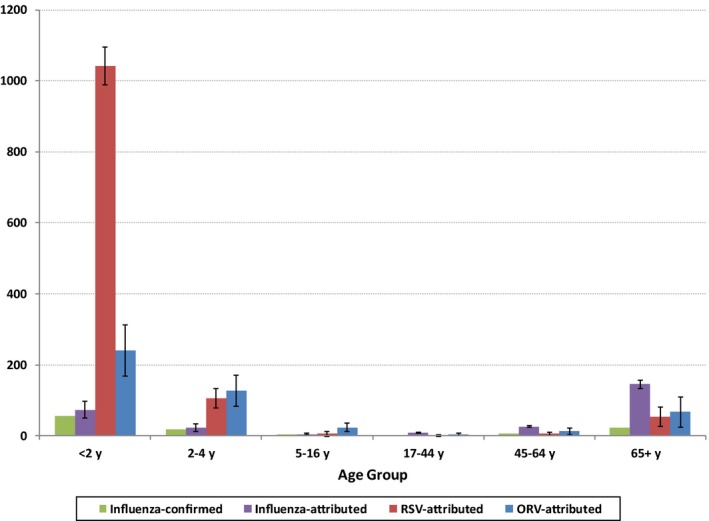
Respiratory hospitalization rates/100 000 population, by age group and viral attribution. The error bars correspond to 95% confidence intervals and are only provided for the estimates of the attributed rates (For data values and additional details, see Table 3)

Over the study period, there was an average of 180 000 hospitalizations for respiratory conditions per year (700 per 100 000 population) of which influenza, RSV, and ORV were identified in 1.1%, 2.6%, and 0.4%, respectively, and attributed to an estimated 4.7%, 5.3%, and 3.8% of respiratory hospitalizations, respectively. For inpatients under the age of 2 years, 25% of primary respiratory hospitalizations had a viral identification (Table 1), with the rate of viral identification declining with increasing age. The average annual rates of excess respiratory hospitalizations attributed to influenza, RSV, and ORV were estimated at 33.1 (95% CI: 29, 38), 27.2 (95% CI: 22, 33), and 27.0 (95% CI: 18, 36) per 100 000 population per year (or approximately 11 000, 9000, and 9000 hospitalizations per year in Canada). Average annual rates were the highest for RSV‐attributed hospitalizations in infants and children under 2 years of age at 1042 (95% CI: 988, 1096) /100 000 infants per year (Figure 1).

**Table 1:** Proportion of respiratory hospitalizations with a viral agent identified, DAD^a^, Canada, 2003/04‐2013/14

Age group (y)	Annual no. of respiratory hospitalizations (×10^3^)	% of Respiratory hospitalizations with a viral identification	% of Respiratory hospitalizations attributable^b^ to a respiratory viral infection
<2	18	25.4	41.7
2‐4	10	7.1	22.1
5‐16	11	2.4	10.8
17‐44	18	1.3	7.2
45‐64	32	1.4	9.3
65+	91	1.2	10.3
Total	180	4.1	13.9

a Discharge Abstract Database (DAD) excludes the province of Quebec.

b Model estimated number of hospitalizations attributed to influenza, RSV, or ORV as a percent of all respiratory hospitalizations by age group.

### 3.1 Influenza‐attributed hospitalizations by diagnostic subgroup

**Figure 2:**
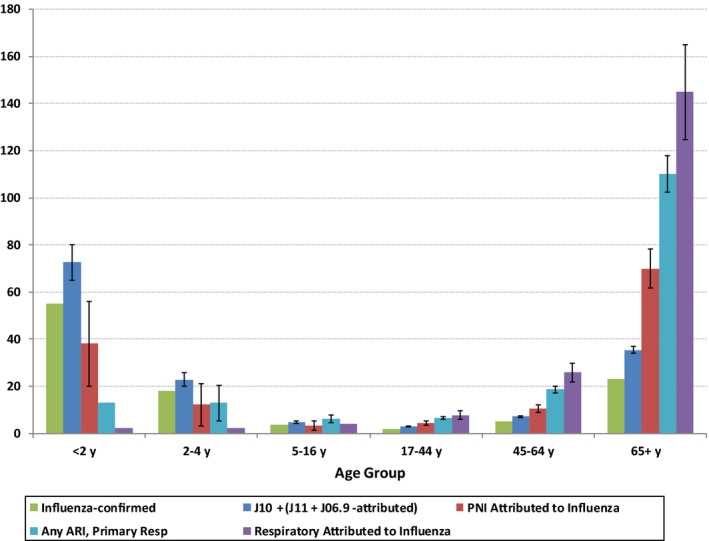
Influenza hospitalization rates/100 000 population and attribution of respiratory hospitalizations to influenza by age group and diagnostic category (For data values and additional details, see Table 2)

Influenza‐attributed hospitalization rates were highest in the oldest and youngest age groups although the impact of the diagnostic subgroup varied with age (Figure 2). In the pediatric population, the only diagnostic group other than influenza (J10) for which we found a notable excess associated with influenza activity was the ILI and other unspecified acute respiratory infection (J11, J06.9) categories estimated at 3.3 (95% CI: 2.7, 3.8) per 100 000 population. The average annual rate of influenza hospitalizations (J10, virus identified) was 11.7/100 000, while the estimated rate of excess influenza‐attributed respiratory hospitalizations was considerably lower at 3.8 (95% CI: −1.6, 9.2) (Table 2). Restricting the category to any ARI resulted in an estimated excess rate of 8.0 (95% CI: 4.3, 11.8). These otherwise apparently contradictory estimates can be explained by the statistically significant offset (or negative excess) for non‐ARI respiratory admissions with the rate estimated at −4.6 (95% CI: −7.0, −2.2)/100 000. The burden attributable to influenza for the pediatric age groups is therefore assumed to be best represented by the sum of influenza hospitalizations (J10) plus the influenza‐attributed ILI and unspecified acute respiratory infection hospitalizations, for an influenza‐attributed rate of 15.0 and an estimated completeness of 78% (95% CI: 75, 81) (Table 3).

**Table 2:** Average annual rates per 100 000 population for influenza and influenza‐attributed hospitalization by diagnostic category and age group, Canada^a^, 2003/04‐2013/14

Age group (y)	Influenza virus identified (J10)	Influenza‐attributed					
		ILI + J06.9^b^ (95% CI)	Primary respiratory (95% CI)	Primary respiratory dx with an ARI^c^ (95% CI)	Primary respiratory dx without ARI (95% CI)	Pneumonia and influenza (J10‐J18) (95% CI)	Proportion of any ARI dx (%)
<2	55.1	17.5 (10, 25)	2.1 (−21, 26)	12.8 (−8.8, 34.4)	−10.5 (−16, −5)	38.0 (20, 56)	na
2‐4	17.9	4.8 (1.9, 7.7)	2.0 (−9.1, 13.1)	12.8 (5.2, 20.4)	−11.7 (−18, −6)	12.1 (3, 21)	na
5‐16	3.7	0.8 (0.3, 1.4)	3.8 (0.8, 6.9)	6.1 (4.6, 7.7)	−1.9 (−3.9, 0.0)	3.2 (1.3, 5.1)	na
17‐44	1.9	1.1 (0.9, 1.3)	7.6 (6.6, 8.6)	6.4 (5.8, 7.1)	1.1 (0.5, 1.7)	4.4 (3.6, 5.3)	85
45‐64	5.2	1.9 (1.6, 2.3)	25.8 (24, 28)	18.7 (17, 20)	7.0 (5.9, 8.2)	10.5 (9, 12)	72
65+	22.9	12.5 (11.2, 13.8)	144.9 (134, 156)	110.1 (102, 118)	34.6 (30, 39)	70.0 (62, 78)	76
All ages	**7.6**	**3.4** (3.2, 3.6)	**31.7** (27, 36)	24.3 (22.5, 26.0)	6.2 (5.1, 7.3)	16.0 (14, 18)	77
Pediatric	11.7	3.3 (2.7, 3.8)	3.8 (−1.6, 9.2)	8.0 (4.3, 11.8)	−4.6 (−7.0, −2.2)	8.5 (6, 11)	na
Adult	6.6	3.4 (3.2, 3.5)	37.6 (32, 43)	28.4 (26.6,30.1)	8.9 (7.8, 10)	17.7 (16, 19)	75

a Quebec does not participate in the DAD (discharge hospital database). The study period excludes 2008/09 and 2009/10 due to the circulation of a novel pandemic strain.

b J11 corresponds to a clinical diagnosis of influenza or ILI; J06.9 corresponds to an unspecified acute respiratory infection.

c ARI, acute respiratory infection, J00‐J22, J44.0

**Table 3:** Viral respiratory hospitalizations 2003/2004‐2013/2014, and respiratory hospitalizations attributable to influenza, RSV, and ORV, average annual rates per 100 000 population, Canada^a^

Age group (y)	Influenza identified	Influenza‐attributed (95% CI)	Completeness^b^ of influenza identification, % (95% CI)
<2	55.1	72.5 (64.9, 80.2)	76 (69, 85)
2‐4	17.9	22.7 (19.8, 25.6)	79 (70, 90)
5‐16	3.7	4.6 (4.0, 5.1)	82 (73, 93)
17‐44	1.9	7.6 (6.6, 8.6)	24 (22, 28)
45‐64	5.2	25.8 (24, 28)	20 (18, 22)
65+	22.9	144.9 (134, 156)	16 (15, 17)
All ages	**7.6**	33.1 (29, 38)	23 (20, 27)
Pediatric^c^	11.7	15.0 (14.4, 15.5)	78 (75, 81)
Adult	6.6	37.6 (32, 43)	17 (15, 21)

Age group (y)	RSV identified	RSV‐attributed (95% CI)	Completeness^b^ of RSV Identification, % (95% CI)
<2	709.3	1042.1 (988, 1096)	68 (65, 72)
2‐4	51.6	106.2 (79, 134)	49 (39, 66)
5‐16	1.9	5.3 (−2.4, 12.9)	36 (15, 100)
17‐44	0.1	1.2 (−1.3, 3.6)	12 (4, 100)
45‐64	0.6	5.0 (−0.2, 10.1)	12 (6, 100)
65+	4.1	52.7 (25, 80)	8 (5, 16)
All ages	**18.2**	**27.2** (22, 33)	**67 (55, 85)**
Pediatric	**87.6**	**133.5** (120, 147)	**66 (60, 73)**
Adult	**1.0**	**11.2** (5, 17)	**9 (6, 19)**

Age group (y)	ORV identified	ORV‐attributed (95% CI)	Completeness^b^ of ORV identification, % (95% CI)
<2	60.5	240.9 (169, 313)	25 (19, 36)
2‐4	13.3	127.3 (84, 171)	10 (8, 16)
5‐16	1.9	23.2 (11.0, 35.5)	8 (5, 17)
17‐44	0.4	3.9 (0.1, 7.7)	9 (5, 429)
45‐64	0.9	12.0 (3.7, 20.3)	7 (4, 25)
65+	3.5	67.2 (25, 110)	5 (3, 14)
All ages	**2.9**	**27.0** (18, 36)	**11 (8, 16)**
Pediatric	**10.2**	**66.4** (47, 86)	**15 (12, 22)**
Adult	**1.1**	**16.5** (12, 21)	**7 (5, 9)**

a Quebec does not participate in the DAD (discharge hospital database). The study period excludes 2008/09 and 2009/10 due to the circulation of a novel pandemic strain.

b Estimated proportion of influenza‐, RSV‐, or ORV‐attributed hospitalizations with viral identification. When the lower 95% CI of the estimated excess was less than the number with virus identified, the upper 95% CI of the completeness estimate was replaced with 100%.

c For the pediatric age groups, influenza‐attributed hospitalizations include those with the influenza virus identified plus excess admissions for J11 (clinical diagnosis of influenza or ILI) and J06.9 (unspecified acute respiratory infections). All other estimates are based on excess hospitalizations with a primary respiratory diagnosis.

For adults, statistically significant excesses were associated with all respiratory subgroups as reported in Table 2. An ARI diagnosis was present in approximately 75% of the influenza‐attributed hospitalizations with estimated rates of 28.4 (95% CI: 26.6, 30.1)/100 000 and 37.6 (95% CI: 32, 43)/100 000 for ARI and all respiratory conditions, respectively (Table 2). The estimated completeness of influenza virus identification was only 17% (95% CI: 15, 21) (Table 3).

### 3.2 RSV‐ and ORV‐attributed hospitalizations

Respiratory syncytial virus was identified in an estimated 66% (95% CI: 60, 73) of excess respiratory admissions attributed to the virus in pediatric patients, though in only 9% (95% CI: 6, 19) for adults. For ORV, viral identification rates were lower at 15% (95% CI: 12, 22) for pediatric patients and 7% (95% CI: 5, 9) for adults. Age‐specific rates for RSV and ORV are provided in Table 3.

### 3.3 Disease severity

An estimated 11% of influenza‐attributed hospitalizations were admitted to a SCU. While influenza was identified in an estimated 23% (95% CI: 20, 27) of the hospitalizations attributed to influenza, and 33% (95% CI: 28, 40) of SCU admissions attributed to influenza, for inpatient deaths, the viral identification rate was lower at 15% (95% CI: 13, 16). For RSV, the rates of viral identification for patients admitted to a SCU were similar to the rates for respiratory hospitalizations. Most RSV hospitalizations occur in infants (<2 years of age) which contributes to the much higher proportion of viral identification among hospitalizations attributed to RSV. Detailed rates for viral identification and viral attribution by status are provided in Table 4.

**Table 4:** Estimated rates and completeness of viral identification among respiratory inpatients by special care status, DAD^a^, Canada, 2003/04‐2013/14

Virus	Status	Virus identified rate/100 000 population	Viral‐attributed^c^ rate/100 000 population (95% CI)	Completeness^d^ of viral identification, % (95% CI)
Influenza	Respiratory hospitalizations	7.6	33.1 (28.6, 37.6)	23 (20, 27)
	Inpatient deaths	0.4	2.6 (2.3, 2.8)	15 (13, 16)
	Special care unit	1.3	3.8 (3.2, 4.5)	33 (28, 40)
RSV^b^	Respiratory Hospitalizations	18.2	27.2 (21.5, 32.9)	67 (55, 85)
	Inpatient deaths	0.1	0.6 (‐.1, 1.3)	11 (5, 100)
	Special care unit	1.6	1.5 (.6, 2.3)	55 (42, 81)
ORV	Respiratory Hospitalizations	2.9	27.0 (17.6, 36.3)	11 (8, 16)
	Inpatient deaths	0.1	0.5 (−0.5, 1.5)	16 (5, 100)
	Special care unit	0.6	1.7 (.2, 3.2)	48 (22, 100)

a Quebec does not participate in the DAD (discharge hospital database). The study period excludes 2008/09 and 2009/10 due to the circulation of a novel pandemic strain.

b Most RSV hospitalizations occur in infants (<2 y of age) which contributes to the much higher proportion of viral identification among hospitalizations attributed to RSV.

c Model estimated excess hospitalizations attributed to the corresponding viral agent.

d When the lower 95% CI of the estimated excess (viral‐attributed) was less the number with the virus identified, the upper 95% CI of the completeness estimate was replaced with 100%.

## 4 INTERPRETATION

This study confirms the very high rates of RSV hospitalizations in young children and that viral identification likely occurs in a majority of the hospitalizations due to influenza or RSV in the pediatric population in Canada. Among adults, influenza likely accounts for more than half of all hospitalizations due to a respiratory virus; however, completeness of viral identification for influenza is much lower among adults, and declines with increasing age. Completeness of influenza viral identification was higher for patients admitted to a special care unit than for inpatient deaths. One‐quarter of respiratory hospitalizations attributed to influenza did not have an ARI diagnosis recorded.

### 4.1 Other Canadian studies and validation

This study updates previous estimates of rates of respiratory hospitalizations attributable to influenza, RSV, and other respiratory viruses^15^, ^16^ for a period when ICD‐9 was used, and more recent studies focused on the completeness of influenza virus identification for the 2009 pandemic^7^ and cross‐validation of annual estimates of morbidity and mortality.^6^

For the ICD‐9 coding period, the weekly number of influenza, RSV, parainfluenza, and adenovirus positive laboratory tests from FluWatch was used as the measure of viral activity.^15^ For the ICD‐10 coding period, we switched to hospitalizations with viral identification as the measure of weekly viral activity as this improved the model fit and resulted in somewhat narrower confidence intervals for the burden estimates.^6^ Although the influenza‐attributed estimates were similar for the 2 approaches, the ecological study design was generally not powerful enough to estimate the burden of 3 other respiratory viruses separately. For the adult age groups, the impact of ORV was consistent enough from year to year that it could be omitted from the regression model as it was accounted for by the seasonal parameters included in the regression model.^6^ Hence, inclusion or exclusion of ORV was not expected to have a marked impact on the estimated rates of influenza‐attributed respiratory hospitalizations. Taking advantage in this study of the ORV identifications coded under ICD‐10, we renewed our efforts to include a measure of the burden for other respiratory viruses with the introduction of a combined ORV category. This approach resulted in reasonable precision.

In our previous studies, we have had similar difficulties estimating the excess influenza burden for pediatric inpatients from all respiratory admissions. The offsetting (or negative) excess associated with influenza activity for non‐ARI respiratory hospitalizations, which we have seen before among non‐respiratory hospitalizations for adults,^15^ suggests that the concept of excess influenza hospitalizations may not be appropriate for the pediatric age groups. However, as we found a statistically significant excess for RSV among non‐ARI respiratory hospitalizations (results not shown), we have likely underestimated the full burden for influenza in the pediatric age group. We detected an excess associated with influenza among the ILI and unspecified acute respiratory infection category (J11, J06.9), so we conclude that the viral identification for influenza is not complete.

### 4.2 Comparison with other studies

American studies suggest a more substantial underdiagnosis of influenza for the US population. A similar regression‐based study by Ortiz et al^20^ found only 1 of 10 influenza‐associated critical illness hospitalizations included an influenza diagnosis (including clinical diagnoses of ILI), compared to our estimate of 1 of 3 for respiratory patients admitted to a special care unit. A US study by Zhou et al^21^ found similar patterns for influenza and RSV, noting that although the overall morbidity burden of influenza and RSV was similar, the age‐specific burdens differ dramatically. Their completeness estimates were similar at 20% and 60% compared to our estimates of 23% and 67% for influenza and RSV, respectively. However, this study was ICD‐9‐based and influenza and RSV diagnoses would have included clinical diagnoses as well as those based on viral identifications. We note that their estimated rates of excess hospitalizations as well as those coded for influenza and RSV were higher than our Canadian results.

A modeling study based on estimates of testing probabilities and test sensitivity by Reed et al^10^ calculated multipliers of 2, 3, and 5 for pediatric age group, working‐age adults, and adults aged 65+, respectively; however, these estimates were for the PNI diagnostic group only. Our estimated influenza multiplier for the pediatric age group of 1.3 (the multiplier is the reciprocal of completeness in Table 2) is lower than the US estimate. From Table 2, we note that influenza was confirmed in 1 of 2, and 1 of 3 influenza‐attributed PNI hospitalizations for working‐age adults and adults aged 65+, respectively. We note that the use of PNI in our study accounted for only half of the estimated hospitalizations attributed to influenza.

Of note, our pediatric multiplier of 1.3 for seasonal influenza is similar to the multiplier of 1.2 estimated for this population in the 2009 pandemic.^7^ However, our adult multipliers for seasonal influenza (which increase with age ranging from 4 to 6) are higher than those estimated for the pandemic (1.5‐2).

### 4.3 Limitations

This is an ecological study design, and the wide confidence intervals for estimates of the rate of excess respiratory hospitalizations attributable to influenza in the pediatric age groups, as well as for other categories, illustrate the limitations of this approach. The relatively small number of excess hospitalizations attributable to influenza compared to RSV among pediatric inpatients contributes to the imprecision.^16^ We have concluded that the ecological estimate of excess respiratory hospitalizations for the pediatric age groups (ie, the influenza‐attributed respiratory estimate) is an underestimate of the true burden due to evidence of an offset (negative excess) in non‐ARI respiratory hospitalizations. As we have seen evidence of a similar offset effect^22^ in non‐respiratory admissions for adults,^15^ it is possible that there was a reduction in other respiratory hospitalizations to accommodate the influenza surge. Our estimates of completeness are only approximate as the ecological estimates of burden are a measure the net excess rather than the number of hospitalizations truly due to the virus. As a result, there will be some discrepancy between this approach and the one used by Reed et al.^10^ Low viral testing rates among diagnostic groups associated with a specific respiratory virus, such as croup and parainfluenza virus,^16^, ^23^ could result in large confidence intervals, or biased results. Noting that a viral identification was recorded in only 2% of croup hospitalizations, we are unsure of whether we have captured the full ORV‐attributed burden. The actual national burden may differ slightly from our estimates as data from the province of Quebec were not available from the DAD. Variation from season to season would likely overshadow regional differences, although completeness of viral identification could vary considerably across provinces, regional health units, or even by hospital, and over time. Hence, the estimates of completeness may not apply to Quebec, or necessarily to any individual province, health unit, or hospital.

As we do not currently have information on which inpatients were tested, we could not evaluate whether the excess influenza‐attributed ILI and unspecified acute respiratory infection hospitalizations correspond to patients for whom testing was not done, or for patients for whom the specimen was test negative. As time since symptom onset and sample procurement and handling can influence the sensitivity of viral identification, it is possible that hospitalizations for which the specimen tested negative were still due to one of the tested viruses.^24^ In which case, the operational sensitivity is likely lower than the manufacturer's estimate of sensitivity as used in the study by Reed et al.^10^

As the ILI diagnostic category is not typically used for patients once the virus is identified, we cannot assess the impact of using only the clinical ILI/SARI diagnostic category for testing purposes as suggested by the WHO sentinel surveillance program recommendations.^4^ The WHO ILI and SARI case definitions are not identical to the ICD‐10 codes for ILI and ARI. The ICD‐10 definition of ARI is much broader than the ICD‐10 ILI definition, while the WHO SARI definition is similar to their ILI case definition (acute respiratory infections with recent onset of cough and fever ≥38°C) but restricted to hospitalized cases. Hence, we were not able to fully assess the potential impact of using a SARI approach on our burden estimates. In a study of emergency department visits where only the clinical or pre‐test diagnoses are recorded, we found that 58% of ILI visits were likely due to influenza, but that an ILI diagnosis was present in only 7% of excess respiratory emergency department visits attributed to seasonal influenza.^8^ The ARI diagnostic category captured 84% of influenza‐attributed ED visits. However, only 10% of all ARI ED visits were likely due to seasonal influenza. Similar results were found in a laboratory‐based study during a period of peak influenza activity.^25^

## 5 CONCLUSIONS

Though overall, the morbidity burden for influenza, RSV, and ORV is similar; the age distribution is very different with the rate of RSV‐attributed hospitalizations among infants particularly high. Testing for the purpose of viral identification is still not fully routine in clinical practice, although viral identification among pediatric inpatients appears to capture most of the hospitalizations attributable to influenza and RSV. FluWatch as well as other influenza surveillance programs now include near real‐time weekly surveillance reports on virologically confirmed hospitalizations. These new datasets and new approaches of measuring the burden of influenza should be compared with the regression‐based estimates of excess burden as a way of assessing representativeness and as an aid to interpreting any differences. When dealing with near real‐time data, delays in reporting make the interpretation of weekly data considerably more complex.^26^, ^27^, ^28^ Use of an ARI case definition to identify cases for viral identification will result in the identification of a much larger proportion of hospitalizations due to influenza than use of an ILI definition, although at a cost of considerably higher testing rates.^25^ An advantage of a sentinel surveillance program is that in capturing the test negative inpatient results, rates can be extrapolated to the full population. Even with a SARI case definition as proposed by the WHO, ensuring representation will be a challenge, with considerable underrepresentation of elderly inpatients with chronic respiratory conditions (such as COPD) expected as symptoms of an acute respiratory infection were either not present or overlooked in our data. For countries with limited or irregular viral identification data, the routine testing of inpatients from sentinel hospitals meeting either the ILI, ARI, or SARI case definitions should provide the quality weekly virological data required for regression‐based estimates of excess mortality and morbidity associated with influenza.

## CONFLICT OF INTEREST

None declared.

## AUTHOR CONTRIBUTIONS

DLS performed the analysis and drafted the manuscript. All contributed to the concept and design of the study and to the interpretation and presentation of study results. All authors revised the manuscript critically and all approved the final version submitted for publication.

## References

[1] World Health Organization (WHO) . Comparing deaths from pandemic and seasonal influenza: pandemic (H1N1) 2009 briefing note 20. 2009 http://www.who.int/csr/disease/swineflu/notes/briefing_20091222/en/index.html. Accessed October 11, 2017.

[2] Thompson WW , Shay DK , Weintraub E , et al. Mortality associated with influenza and respiratory syncytial virus in the United States. JAMA. 2003;289:179‐186. 12517228 10.1001/jama.289.2.179

[3] Simonsen L , Spreeuwenberg P , Lustig R , et al. Global mortality estimates for the 2009 influenza pandemic from the GLaMOR project: a modeling study. PLoS Med. 2013;10:e1001558. 24302890 10.1371/journal.pmed.1001558 PMC3841239

[4] World Health Organization . Global epidemiological surveillance standards for influenza. 2014 http://www.who.int/influenza/resources/documents/WHO_Epidemiological_Influenza_Surveillance_Standards_2014.pdf?ua=1. Accessed October 11, 2017.

[5] Thompson WW , Shay DK , Weintraub E , et al. Influenza‐associated hospitalizations in the United States. JAMA. 2004;292:1333‐1340. 15367555 10.1001/jama.292.11.1333

[6] Schanzer DL , Sevenhuysen C , Winchester B , Mersereau T . Estimating influenza deaths in Canada, 1992‐2009. PLoS One. 2013;8:e80481. 24312225 10.1371/journal.pone.0080481 PMC3842334

[7] Schanzer DL , McGeer A , Morris K . Statistical estimates of hospital admissions attributable to seasonal and pandemic influenza for Canada. Influenza Other Respir Viruses. 2012;7:799‐808. 23122189 10.1111/irv.12011 PMC3796862

[8] Schanzer DL , Schwartz B . Impact of seasonal and pandemic influenza on emergency department visits, 2003‐2010, Ontario, Canada. Acad Emerg Med. 2013;20:388‐397. 23701347 10.1111/acem.12111 PMC3748786

[9] Schanzer D , Zheng H , Gilmore J . Statistical estimates of absenteeism attributable to influenza from the labour force survey. BMC Infect Dis. 2011;11:1‐9. 21486453 10.1186/1471-2334-11-90 PMC3103439

[10] Reed C , Chaves SS , Kirley PD , et al. Estimating influenza disease burden from population‐based surveillance data in the United States. PLoS One. 2015;10:e0118369 https://doi.org/10.1371/journal.pone.0118369. 25738736 10.1371/journal.pone.0118369 PMC4349859

[11] Goldstein E , Viboud C , Charu V , Lipsitch M . Improving the estimation of influenza‐related mortality over a seasonal baseline. Epidemiology. 2012;23:829‐838. 22992574 10.1097/EDE.0b013e31826c2dda PMC3516362

[12] Thompson WW , Weintraub E , Dhankhar P , et al. Estimates of US influenza‐associated deaths made using four different methods. Influenza Other Respi Viruses. 2009;3:37‐49. 10.1111/j.1750-2659.2009.00073.x PMC4986622 19453440

[13] Yang L , Chiu SS , Chan KP , et al. Validation of statistical models for estimating hospitalization associated with influenza and other respiratory viruses. PLoS One. 2011;6:e17882. 21412433 10.1371/journal.pone.0017882 PMC3055891

[14] Muscatello DJ , Newall AT , Dwyer DE , MacIntyre CR . Mortality attributable to seasonal and pandemic influenza, Australia, 2003 to 2009, using a novel time series smoothing approach. PLoS One. 2013;8:e64734 https://doi.org/10.1371/journal.pone.0064734. 23755139 10.1371/journal.pone.0064734 PMC3670851

[15] Schanzer DL , Langley JM , Tam TWS . Role of influenza and other respiratory viruses in admissions of adults to Canadian hospitals. Influenza Other Respir Viruses. 2008;2:1‐8. 19453488 10.1111/j.1750-2659.2008.00035.x PMC4634329

[16] Schanzer DL , Langley JM , Tam TWS . Hospitalization attributable to influenza and other viral respiratory illnesses in Canadian children. Pediatr Infect Dis J. 2006;25:795‐800. 16940836 10.1097/01.inf.0000232632.86800.8c

[17] Canadian Institute for Health Information . Discharge abstract database (DAD) metadata. https://www.cihi.ca/en/dadnacrs-abstracting-manual-web-tool. Accessed October 11, 2017.

[18] World Health Organization (WHO) . ICD‐10: International statistical classification of diseases and related health problems, tenth revision. http://apps.who.int/classifications/icd10/browse/2010/en. Accessed October 11, 2017.

[19] Statistics Canada . Table 051‐0001 – Estimates of population, by age group and sex for July 1, Canada, provinces and territories, annual (persons unless otherwise noted), CANSIM (database). http://www.statcan.gc.ca/tables-tableaux/sum-som/l01/cst01/demo31a-eng.htm. Accessed October 11, 2017.

[20] Ortiz JR , Neuzil KM , Shay DK , et al. The burden of influenza‐associated critical illness hospitalizations. Crit Care Med. 2014;42:2325‐2332. 25148596 10.1097/CCM.0000000000000545 PMC4620028

[21] Zhou H , Thompson WW , Viboud CG , et al. Hospitalizations associated with influenza and respiratory syncytial virus in the United States, 1993‐2008. Clin Infect Dis. 2012;54:1427‐1436. 22495079 10.1093/cid/cis211 PMC3334364

[22] Canadian Institute for Health Information . The impact of the H1N1 pandemic on Canadian hospitals. https://secure.cihi.ca/free_products/H1N1_AIB_final_EN.pdf. Accessed October 11, 2017.

[23] Iwane MK , Edwards KM , Szilagyi PG , et al. Population‐based surveillance for hospitalizations associated with respiratory syncytial virus, influenza virus, and parainfluenza viruses among young children. Pediatrics. 2004;113:1758‐1764. 15173503 10.1542/peds.113.6.1758

[24] Schanzer DL , Garner MJ , Hatchette TF , Langley JM , Aziz S , Tam TWS . Estimating sensitivity of laboratory testing for influenza in Canada through modelling. PLoS One. 2009;4:e6681. 19688094 10.1371/journal.pone.0006681 PMC2722738

[25] Amini R , Gilca R , Douville‐Fradet M , Boulianne N , De Serres G . Evaluation of the new World Health Organization case definition of severe acute respiratory infection for influenza surveillance during the peak weeks of two influenza seasons in Quebec, Canada. J Pediatr Infect Dis Soc. 2016; 6:297‐300. 10.1093/jpids/piw044 27496537

[26] Hsieh Y , Fisman DN , Wu J . On epidemic modeling in real time: An application to the 2009 novel A (H1N1) influenza outbreak in Canada. BMC Res Notes. 2010;3:283 https://doi.org/10.1186/1756-0500-3-283. 21050494 10.1186/1756-0500-3-283 PMC2989981

[27] Azmon A , Faes C , Hens N . On the estimation of the reproduction number based on misreported epidemic data. Stat Med. 2014;33:1176‐1192. 24122943 10.1002/sim.6015

[28] Lawless JF . Adjustments for reporting delays and the prediction of occurred but not reported events. Can J Stat. 1994;22:15‐31.

