# Supplementary material for: Burden of influenza, respiratory syncytial virus, and other respiratory viruses and the completeness of respiratory viral identification among respiratory inpatients, Canada, 2003‐2014
Source: Influenza Other Respir Viruses. 2017 Dec 15;12(1):113–21. doi: 10.1111/irv.12497 (PMC5818333; doi:10.1111/irv.12497)
Supplement: Supplementary file 1 [file IRV-12-113-s001.docx]

Appendix S1, Supplementary Table:

|  | **ICD-10 code** | **Definition** | **Group Label** |
| --- | --- | --- | --- |
| *Measures of viral activity/ Number of hospitalizations with viral identification* | | | |
|  | B34.0-B34.4 | Other Viral infection identified, site unspecified | ORV |
|  | B97.0-B97.3, B97.5-B97.7 | Other viral agents as the cause of diseases classified to other chapters | ORV |
|  | B97.4 | RSV as the cause of diseases classified to other chapters | RSV |
|  | J10 | Influenza virus identified | Influenza |
|  | J12.1 | Viral pneumonia, RSV | RSV |
|  | J20.5 | Acute bronchitis due to RSV | RSV |
|  | J21.0 | Acute bronchiolitis due to RSV | RSV |
|  | J12.0, J12.2-J12.8 | Viral pneumonia due to other specified viruses | ORV |
|  | J20.0-J20.4, J20.6-J20.7 | Acute bronchitis due to other specified viruses | ORV |
|  | J21.1 | Acute bronchiolitis due other specified viruses (human metapneumovirus) | ORV |
| *Dependent variables for the regression model* | | |  |
|  | J00-J99 | Diseases of the respiratory system | Respiratory |
|  | J00-J22, J44.0 | Acute Respiratory Infection (ARI) | ARI |
|  | J06.9 | Unspecified acute respiratory infection | ILI + J06.9 |
|  | J11 | Influenza-like illness, virus not identified | ILI + J06.9 |
|  | J10-J18 | Pneumonia and influenza | PNI |

Appendix S2: Quasi-Poisson regression model

The regression model was fit using SAS 9.1 ^1^ PROC GENMOD with a Poisson distribution, linear link function and dispersion parameter specified by:

where ***NAdms*** represents the weekly number of admissions to hospital for the category of interest (for example, all respiratory or pneumonia and influenza or specific age group). The monthly indicator variables (*Mon_m_*) and the sinusoidal terms (with *t*= 2π *week*/52.177457) are included to account for weekly patterns due to seasonality. *FY_y_* is a set of indicator variables for each season, *y,* starting in September. The 2008/09 and 2009/10 seasons were excluded due to the pandemic. The *β*_4_ parameters account a general trend, and force the residuals (ε) to sum to zero over each flu year (*FY*). The variables ***Holiday*** and ***Dec25*** were included to account for the potential effects of holidays when admissions are generally lower, and in particular the last week of December (that includes December 25^th^). The variable ***Jan1*** and ***Sept1*** are indicator variables for the first week of January and first week of September, to account for spikes seen in respiratory admissions and asthma admissions in some age groups. The *β*_9,_ *β*_10,_*_y_*_,_ and *β*_11,_ *_y_* parameters are multipliers for the proxy variables for ORV, influenza and RSV respectively. Separate annual parameters for each flu season were included to account for annual variation in completeness of viral identification. The indicator variable FY is included as interaction term to indicate that, for example, *β*_10, 2005_ applies only to Inflproxy for the FY of 2005. The use of separate annual parameters is essential to produce independent annual estimates of the excess burden (with a single parameter, the annual estimates will be perfectly correlated with the proxy variable for viral activity). Unfortunately, statistical power was insufficient to estimate the annual effects of ORV, and RSV for some age groups. As annual estimates have not been reported, in most cases the FY interaction is not needed. This can be easily confirmed by running both versions. The impact of dropping the “FY*” term (i.e., replacing the *β*_10,_*_y_*_,_ and *β*_11,_ *_y_* parameters by a single parameter *β*_10_ and *β*_11_) was assessed and usually found to have a minimal impact on the average annual attributed rates (see ^2^ for more details on the effect of parameterization). The ***Fluproxy*** is the weekly number of hospitalizations with the influenza virus identified (J10); the ***RSVproxy*** variable is the weekly number of hospitalizations with the RSV virus identified (J12.1, J20.5, J21.0, B97.4) and the ***ORVproxy*** variable is the weekly number of hospitalizations with another respiratory virus specified (J12.0, J12.2-J12.8, J20.0-J20.4, J20.6-J20.7, J21.1, B34.0-B34.4, B97.0-B97.7). The dispersion parameter was included in the model estimation to account for additional variation due to events not captured by the explanatory variables and usually results in larger confidence intervals.

The number of weekly hospitalizations attributed to each virus was calculated as the difference between model-predicted number of hospitalizations and the model-predicted hospitalizations under the hypothetical absence of the corresponding virus. The model-predicted number of hospitalizations under the hypothetical absence of one or all three viruses is referred to as a seasonal baseline. The number of hospitalizations attributed to each variable can also be calculated directly from the regression equation above as

where the proxy variable is summed over the regression period or season according to the regression equation used for estimation. The former calculation facilitates calculation of a weekly baseline for a graphical presentation, while this latter calculation corresponds to the approach used for calculating confidence intervals. Confidence intervals for estimates of influenza-attributed rates were calculated from the coefficient of variation of the corresponding parameter (*β*_9,_ *β*_10,_ and *β*_11_).

References:

1. SAS Institute Inc. SAS Enterprise Guide 5.1. 2015. Available: https://support.sas.com/documentation/onlinedoc/guide/.

2. Schanzer DL, Sevenhuysen C, Winchester B, Mersereau T. Estimating influenza deaths in Canada, 1992-2009. PLoS One. 2013;8: e80481. doi: 10.1371/journal.pone.0080481. Available: <http://journals.plos.org/plosone/article?id=10.1371/journal.pone.0080481>.
